# The Role of Omics Approaches to Characterize Molecular Mechanisms of Rare Ovarian Cancers: Recent Advances and Future Perspectives

**DOI:** 10.3390/biomedicines9101481

**Published:** 2021-10-15

**Authors:** Yashwanth Subbannayya, Riccardo Di Fiore, Silvana Anna Maria Urru, Jean Calleja-Agius

**Affiliations:** 1 Centre of Molecular Inflammation Research (CEMIR), Department of Clinical and Molecular Medicine (IKOM), Norwegian University of Science and Technology, 7491 Trondheim, Norway; 2 Department of Anatomy, Faculty of Medicine and Surgery, University of Malta, MSD 2080 Msida, Malta; riccardo.difiore@um.edu.mt; 3 Sbarro Institute for Cancer Research and Molecular Medicine, Center for Biotechnology, College of Science and Technology, Temple University, Philadelphia, PA 19122, USA; 4 Hospital Pharmacy Unit, Trento General Hospital, Autonomous Province of Trento, 38122 Trento, Italy; silvanaurru@gmail.com; 5 Department of Chemistry and Pharmacy, School of Hospital Pharmacy, University of Sassari, 07100 Sassari, Italy

**Keywords:** systems biology, rare ovarian cancers, precision medicine, multi-omics, data integration, oncology

## Abstract

Rare ovarian cancers are ovarian cancers with an annual incidence of less than 6 cases per 100,000 women. They generally have a poor prognosis due to being delayed diagnosis and treatment. Exploration of molecular mechanisms in these cancers has been challenging due to their rarity and research efforts being fragmented across the world. Omics approaches can provide detailed molecular snapshots of the underlying mechanisms of these cancers. Omics approaches, including genomics, transcriptomics, proteomics, and metabolomics, can identify potential candidate biomarkers for diagnosis, prognosis, and screening of rare gynecological cancers and can aid in identifying therapeutic targets. The integration of multiple omics techniques using approaches such as proteogenomics can provide a detailed understanding of the molecular mechanisms of carcinogenesis and cancer progression. Further, omics approaches can provide clues towards developing immunotherapies, cancer recurrence, and drug resistance in tumors; and form a platform for personalized medicine. The current review focuses on the application of omics approaches and integrative biology to gain a better understanding of rare ovarian cancers.

## 1. Introduction

Ovarian cancer is one of the most lethal gynecological malignancies [1]. The contributing factors to its poor prognosis include the fact that its initial symptoms are not always obvious, making it difficult for early detection. Hence, at the time of diagnosis, most patients are already at least at Stage 3, leading to a survival rate of about 50% [2,3]. Moreover, compared with other gynecological cancers, ovarian cancer has a higher recurrence rate, leading to a poorer cure rate. In addition, almost half of the ovarian malignancies are considered ‘rare’, with an incidence of <6/100,000 women per year [4].

Rare ovarian cancers overall have the same problem of late diagnosis and are therefore more difficult to treat. In addition, they can be misdiagnosed due to their rarity and consequent clinical inexperience [3,5]. This results in poor outcomes and stresses the need for reliable markers for early diagnosis and potential specific therapeutic targets.

The development of next-generation sequencing (NGS) and high-resolution mass spectrometry technologies have enabled large-scale, cost-efficient, multiple ‘omics’ analyses, including genomic, epigenomic, transcriptomic, proteomic, and metabolomic research [6,7]. In cancer, omics technologies have a wide range of applications in both basic research and clinical treatment. Using NGS, genomics and transcriptomics provide a better understanding of the structure of the cancer genome and aid in the discovery of differentially expressed genes that drive and maintain tumorigenesis [7]. Moreover, omics-based profiling can establish different molecular subtypes, which is crucial for personalized therapies.

However, single-level omics approaches are limited by the lack of resolving power to establish causal relationships between phenotypic manifestations and molecular alterations [8]. In contrast, systems biology integrates multidisciplinary information and can deepen the understanding of biological interactions systematically and holistically [8]. Integrating regulatory layers could be especially suitable for dissecting aberrant cellular functions in cancer and other complex diseases [8]. Measuring biological samples on multiple omics scales allows a better understanding of how the interaction between genetic variants and the environment affects biological systems. Multi-omics data analysis, based on machine learning techniques, provides a greater understanding of predictive and prognostic phenotypes, improves the clustering of samples into specific biologically meaningful groups, analyzes the cellular responses to therapy, and contributes to translational research using integrative models [9,10].

This review article describes fundamental principles, challenges, advances, and clinical applications of different “omics” technologies, including genomics, transcriptomics, proteomics, and metabolomics, highlighting the significance of integrating multi-omics data specifically in rare ovarian cancer research and evaluating clinically relevant outcomes.

## 2. Ovarian Cancer

Ovarian cancer can be divided into epithelial and non-epithelial types. The most common group of ovarian malignancies are epithelial cancers. These are classified histologically into serous (low and high grade), mucinous, clear cell, endometrioid, carcinosarcoma, and primary peritoneal cancer [11,12]. Specifically, clear-cell, mucinous and low-grade serous carcinoma are considered as rare ovarian malignancies [11,12].

### 2.1. Epithelial Ovarian Cancers

#### 2.1.1. Serous Carcinomas

Low-grade serous ovarian carcinomas (LGSOC) and high-grade serous ovarian carcinomas (HGSOC) may arise from the fallopian tube. Although most patients with low-grade serous carcinoma present at an early stage, they do occasionally present at an advanced stage, and in this case, although the initial treatment may lead to a complete response, relapse is common with poor response to chemotherapy [13]. Low-grade serous carcinomas are often characterized by BRAF, KRAS, NRAS, EIF1AX, and USP9X mutations [14].

#### 2.1.2. Ovarian Clear Cell Cancer

Ovarian clear cell cancer (OCCC), when diagnosed at an earlier stage, tends to have a good prognosis with surgery often being curative. However, in advanced stages, it is more likely to be chemo-resistant and has a worse prognosis [15]. There are similarities in molecular pathways between OCCC and clear-cell carcinoma of the kidney [16], where inhibition of growth-factor signaling, angiogenesis, and mTOR pathways, might contribute to improved survival rates. Treatments such as multikinase inhibitors (sunitinib, sorafenib, axitinib, and pazopanib), bevacizumab, temsirolimus, and everolimus, which are used in the case of clear-cell carcinoma of the kidney, might also have a similar anti-tumor effect in OCCC. However, there are limited preliminary clinical data from studies focusing specifically on OCCC [17]. The commonest somatic genetic alterations in OCCC are: loss of ARID1A (66.7%), activation of PIK3CA (50%), mutations in PPP2R1A (18.8%) and KRAS (16.7%) [18]. Novel treatment strategies for OCCC with ARID1A mutations include the administration of dasatinib and/or the HDAC6 inhibitor ACY1215 and the inhibition of the methyltransferase EZH2 [19,20,21]. ARID1A-mutated OCCC cells are specifically sensitive to small molecule inhibitors of the bromodomain and extra terminal domain (BET) family of proteins, including BRD2. This, in turn, causes a reduction in the expression of multiple SWI/SNF members, including ARID1B [22]. Since expression of epidermal growth factor receptor (EGFR) is detected in up to 60% of OCCCs, EGFR inhibitors have the potential to be effective therapeutic agents [23]. In addition, both in early- and advanced-stage OCCC, there is a high expression of mTOR. Therefore, mTOR inhibitors also have therapeutic potential, particularly in recurrent OCCC with cisplatin resistance [24].

#### 2.1.3. Mucinous Epithelial Ovarian Cancer

Primary mucinous epithelial ovarian cancer (mEOC) constitutes less than 5% of epithelial ovarian cancers (EOCs), with a decreasing incidence due to better diagnosis. In fact, many cases which were previously diagnosed as primary mEOCs were actually metastases from the gastrointestinal tract or other organs. This highlights the importance of clinico-pathological review because very often, pathological assessment does not necessarily distinguish between primary and metastatic mucinous carcinomas. While the recommended treatment of mEOC is using adjuvant carboplatin and paclitaxel, trastuzumab (Herceptin) and HER2- targeted therapies might also be effective as HER2 is amplified in 18.2% [25] or 19% of these tumors [15], respectively.

### 2.2. Non-Epithelial Ovarian Cancers

Non-epithelial ovarian cancers, including sex-cord stromal tumors and malignant germ-cell tumors, are very rare, accounting for only 6% of all ovarian malignancies [26,27,28,29,30].

#### 2.2.1. Malignant Germ Cell Tumors

Malignant germ-cell tumors occur more commonly in women younger than 20 years and are often treated in a similar way as their testicular counterparts. These tumors can be histologically classified as dysgerminoma, immature teratoma, malignant struma ovarii, embryonal carcinoma, choriocarcinoma, yolk sac tumor, mixed germ-cell tumor, gonadoblastoma, and teratoma with malignant transformation [4]. Using platinum-based regimens, the estimated five-year overall survival is above 90% for early-stage tumors and around 75% for advanced disease [28]. Adjuvant chemotherapy using regimens such as bleomycin, etoposide, and cisplatin (BEP) is also in routine use. However, unlike in the case of testicular germ-cell tumors, there are no trials to date to suggest whether, in the case of relapsed ovarian germ-cell tumors, there may be any benefit of a second-line therapy or the utility of high-dose chemotherapy followed by autologous stem cell transplant. Current therapeutic management includes the administration of TIP (paclitaxel, ifosfamide, and cisplatin), based on treatments used for testicular germ-cell tumors, and also other complex regimens with a combination of cisplatin, bleomycin methotrexate, and vincristine, alternating with actinomycin D, cyclophosphamide, and etoposide [27]. Furthermore, other targeted therapies that have been investigated include tyrosine kinase inhibitors (TKIs) (i.e., sunitinib and imatinib), antiangiogenic agents such as thalidomide and bevacizumab, and trastuzumab (anti-HER2 monoclonal antibody) [31].

#### 2.2.2. Granulosa Cell Tumors

Granulosa cell tumors are a type of malignant sex-cord stromal tumor, constituting about 5% of ovarian malignancies. There are two main different types: adult and juvenile granulosa cell tumors. Sertoli-Leydig cell tumors, gynandroblastomas, steroid cell tumors, and sex cord tumors with annular tubules are rare. Histologically, granulosa cell tumors consist of granulosa cells that secrete progesterone and estrogen [31]. In the diagnosis of adult-type tumors, where morphological appearances are not characteristic, it is helpful to test for the C134W FOXL2 mutation [32]. Granulosa cell tumors tend to progress slowly with late recurrence. In patients with advanced-stage or recurrent granulosa cell tumors, traditional chemotherapy has a limited effect [33]. The currently ongoing GOG264 trial (NCT01042522) aims to compare the efficacy of BEP versus carboplatin and paclitaxel in patients with advanced or recurrent sex cord-ovarian stromal cell tumors. Furthermore, targeted therapies such as TKIs, vascular endothelial growth factor (VEGF) inhibitors, and hormonal treatment have been investigated as potential treatment options for granulosa cell tumors [15].

## 3. “Omics” Approaches and Integrative Biology

While several omics approaches currently exist for delineating molecular mechanisms of biological processes, the current review focuses mainly on genomics, transcriptomics, proteomics, and metabolomics approaches employed to investigate rare ovarian cancers. A partial list of these studies is provided in Table 1.

### 3.1. Genomics Approaches for the Identification of Alterations in the Rare Ovarian Cancer Genome

Genomic technologies have led to breakthroughs towards understanding and treating various diseases, including cancers. Technologies ranging from polymerase chain reaction (PCR) to next-generation sequencing (NGS) of cancer genomes and exomes have enabled the identification of drivers of oncogenic processes. In addition, NGS technologies have had a profound impact on genome-wide association studies (GWAS). The genetics of ovarian cancers is a rapidly evolving field that has massive implications on the classification, diagnosis, prognosis, and treatment. Ovarian cancers can be stratified into different clinical subtypes, and extensive diversity is observed with respect to genetics and progression within each subtype. Each clinical subtype behaves differently, and heterogeneity within specific subclasses present challenges regarding treatment options, drug resistance, and overall clinical response. The most common type of ovarian cancer is the HGSOC where TP53 mutations occur in over 90% of all patients [45]. Further, NGS and other molecular investigations have reported mutations in DNA repair pathways, constituting BRCA1 and BRCA2, in about 50% of all high-grade serous patients. This led to the development of therapies involving poly (ADP-ribose) polymerase (PARP) inhibitors [46]. Other than TP53 and BRCA mutations (approximately 10–12% of ovarian cancers), only a small percentage of HGSOCs were found to possess a specific causative mutation that could be targeted therapeutically. Therefore, the implementation of genomic-based medicine remains a challenge for the management of women with ovarian cancer.

Earlier, studies on the genomic landscapes of ovarian cancer subtypes, including efforts by large consortiums such as The Cancer Genome Atlas (TCGA), were predominantly focused on prevalent subtypes. However, several studies exploring the genome of rare ovarian cancers, including clear cell carcinoma, mucinous carcinoma, LGSOC, among others, have since been published, and several genomic alterations in rare ovarian cancers have since been identified (Table 2).

Whole-genome sequencing analysis of OCCC samples by Itamochi and colleagues identified prevalent mutations in ARID1A (42%), PIK3CA (35%), HLA-DRB1 (25%), MUC4 (22%), ZNF717 (22%), and ARID1B (18%) that correlated with improved overall survival in OCCC patients [34]. In addition, activating alterations in genes belonging to the PI3K/AKT signaling pathway correlated with improved patient survival. Jones and colleagues carried out exome sequencing of OCCC samples and identified mutations in PPP2R1A and ARID1A genes [47]. Shibuya and colleagues used an exome sequencing approach to identify frequent mutations of ARID1A, PIK3CA, PPP2R1A, and KRAS in OCCC [18]. An NGS profiling study of mucinous ovarian carcinomas targeted genes commonly mutated in cancers (hotspot mutations) and identified frequent mutations in KRAS and TP53 genes in a majority of the cases [48]. Another study on the mutational landscape of mucinous ovarian cancers used the exome sequencing approach to identify mutations in KRAS, BRAF, CDKN2A, TP53, RNF43, ELF3, GNAS, ERBB3, and KLF5 genes [35]. The authors concluded that the observed diversity of mutations are indicative of multiple tumorigenesis pathways in mucinous ovarian cancers. A whole-genome sequencing analysis of a larger cohort of mucinous ovarian tumors identified frequent alterations in CDKN2A, KRAS and TP53, and ERBB2 genes [49]. Hunter and colleagues performed a genome-wide genomic copy number analysis, mutation hotspot screening, and whole-exome sequencing analysis of low-grade serous ovarian cancers [50]. They found RAS/RAF/ERBB2 mutations in 63% of LGSOCs and identified BRAF, KRAS, NRAS, USP9X, and EIF1AX genes to be frequently mutated. Targeted sequencing of LGSOC samples identified prevalent hotspot mutations in RAS/RAF signaling genes, including KRAS, BRAF, and NRAS [36]. In addition, frequent USP9X mutations were observed in LGSOC, and further investigations indicated USP9X to be a tumor suppressor gene and a potential therapeutic target for LGSOC.

### 3.2. Exploration of the Rare Ovarian Cancer Transcriptome

Transcriptomics refers to the high-throughput characterization of all RNA molecules in a cell, tissue, or organism. The transcriptome consists of both the protein-coding mRNAs and non-coding RNAs (ncRNA) such as transfer RNAs (tRNAs), ribosomal RNAs (rRNAs), small nuclear RNAs (snRNAs), small nucleolar RNAs (snoRNAs), microRNAs (miRNAs), small interfering RNAs (siRNAs), P-element-induced wimpy testis-interacting (PIWI) RNAs (piRNAs), and long non-coding RNAs (lncRNAs). While micro-array-based gene expression studies were earlier extensively used to characterize the transcriptome, RNA sequencing (or RNA-Seq) has become the primary way to study the transcriptome. Several studies using transcriptomics to study molecular mechanisms of various cancers, including rare ovarian cancers, have been published. A previous study by Wang and colleagues carried out microarray-based gene expression analysis for a cohort of invasive rare EOCs including endometroid, clear cell, mucinous and low-grade cancers to identify expression signatures correlating with the outcome [51]. Cancers with better outcomes showed upregulation of genes involved in steroid hormone biosynthesis and the WNT signaling pathway.

Among the various rare ovarian cancers, the transcriptome of OCCC is the best studied. Several studies have used RNA-Seq to differentiate between OCCC and other cancer types. For example, A previous study exploring differences between OCCC and uterine clear cell carcinomas (UCCC) found that 1607 genes were significantly upregulated and 109 genes were significantly downregulated in OCCC compared to UCCC [52]. Another study by Nagasawa and colleagues employed RNA-sequencing to identify fundamental differences between HGSOC, a common ovarian cancer subtype, with clear cell carcinoma [37]. This study identified that CPNE8 and BHLHE41 genes were characteristic of OCCC and HGSOC, respectively, and could be potentially used as markers for differential diagnosis. Further, RNA-Seq analysis of OVTOKO (ovarian clear cell carcinoma) cell spheroids found that the focal adhesion pathway was essential in spheroids and could serve as a potential therapeutic target for clear cell carcinoma [53]. Further analysis found that treatment of clear cell carcinoma cells with a FAK inhibitor could inhibit the Akt-mTOR pathway. Gene fusions play an important role in several cancers and are potentially druggable. RNA-Seq analysis of EOCs by Earp and colleagues identified that clear cell carcinomas harbored more fusions than other EOC histological types [54]. The fusion gene UBAP1-TGM7 was found to be present in a fraction of clear cell carcinomas.

Ovarian granulosa cell tumors (GCTs) have also been investigated using transcriptomics approaches. Alexiadis and colleagues examined the differences between early-stage and advanced stages of GCTs using a gene expression approach [38]. Transcriptome profiles indicated that 24 genes, including CXCL14, MFAP5, IGF2, and DES (Desmin), were differentially expressed between early and late GCTs. Another study investigating the gene expression profiles of adult ovarian GCTs using a genomics approach identified differential expression of genes related to cell proliferation and cell death [55]. In addition, differentially expressed genes were found to be enriched for FOXL2 target genes in line with previous findings of prevalent FOXL2 somatic mutations in GCTs. A study carried out RNA-Seq of Juvenile granulosa cell tumors and found that genes belonging to the cytokine/hormone signaling and cell division-related processes were differentially regulated [56]. Another study sought to delineate the role of miRNA-10a, involved in the normal development of granulosa cells, in granulosa cell tumors using RNA-Seq [57]. RNA-Seq of miRNA-10a-overexpressed KGN granulosa tumor cells identified miR-NA-10a to be associated with cancer-related pathways such as PI3K-Akt and NFκB signaling.

Single-cell RNA-seq (scRNA-seq) is a transcriptomics approach that can achieve qualitative and quantitative analyses of cell populations in complex tissues without a priori knowledge of cell compositions. As opposed to bulk gene expression (RNA-Seq), scRNA-Seq effectively dissects the tumor transcriptome at single-cell resolution. scRNA-seq datasets can provide several useful information, including (i) identification of cell types in cancers (ii) distinguishing between neoplastic and non-neoplastic cells, (iii) infer signaling mechanisms from gene expression of signaling components (iv) estimating cell types and proportions in bulk gene expression datasets, and (v) characterizing transcriptional dynamics [58]. There are limited scRNA-Seq-based studies on rare ovarian cancers, and there is a need for further scRNA-Seq investigations. An scRNA-Seq-based approach was used to investigate cell population differences in differing grades of serous epithelial ovarian tumors, which included both benign, HGSOC and LGSOC [39]. The study was able to identify sixteen distinct cell populations with specific cells correlated to high-grade, low-grade, and benign tumors. However, there were no distinct tumor epithelial gene expression profiles for both HGSOC and LGSOC.

### 3.3. Interrogation of the Proteomic Landscape of Rare Ovarian Cancers Using Prote-Omics

Proteomics refers to the high-throughput analysis of the entire protein complement of a system such as a cell, tissue, or organism. Proteomic approaches are complementary to genomics approaches as they represent gene products that are active players of molecular mechanisms in cells [59]. Proteomics approaches have been widely used to study biological mechanisms of cancers. The applications of proteomics to cancer biology include identification of markers for cancer diagnosis, monitoring progress, and identification of potential therapeutic targets [60]. Cancer proteomic studies have used a wide array of sample types, including tissue [61], urine [62], serum [63], cell lines [64], secretome [65], and xenografts [66], amongst others to study quantitative changes in protein expression. Proteomics approaches employ several biochemical methods, including mass spectrometry, two-dimensional gel electrophoresis (2D-GE), and reverse-phase protein arrays (RPPA). Proteomics approaches based on high-resolution mass spectrometry can quantitate proteins over an extensive dynamic range ranging from milligrams to a few picograms. This versatility of proteomics has resulted in its frequent use in cancer biology.

The use of proteomics approaches to study rare ovarian cancers has been gradually increasing over the years. Toyama and colleagues carried out a two-dimensional gel electrophoresis-based proteomic characterization of ovarian cancer histological subtypes, including clear cell, endometrioid, mucinous, and serous carcinomas [67]. This resulted in the identification of 55 differential proteins, of which three proteins were able to distinguish between these subtypes. A multi-omics study encompassing cell metabolome, lipidome, proteome, and kinome approaches was used to study differences in drug responses of fatty-acid (FA)-synthase (FASN) blockade in clear cell (SKOV3 cell line) and serous ovarian cancers (OVCAR3 cell line) [68]. Both these subtypes were found to possess disparate metabolic makeup, with SKOV3 favoring glycolysis while OVCAR3 was favoring glycolysis.

Proteomics has also been used to identify differential markers for rare ovarian cancers. A two-dimensional gel electrophoresis and mass spectrometry-based proteomics approach was used by Morita and colleagues to identify potential distinguishing markers between clear cell adenocarcinoma and mucinous adenocarcinoma [40]. A previous study used conditioned media (secretome) from clear cell carcinoma cell lines and other types of EOCs to identify tissue factor pathway inhibitor 2 (TFPI2) as a potential diagnostic marker for differential diagnosis [69]. A follow-up study using patient serum samples validated TFPI2 as a bonafide marker for ovarian clear cell carcinoma showing high levels of sensitivity, specificity, and accuracy [70]. A study by Fata and colleagues employed a proteomic approach using matrix-assisted laser desorption/ionization (MALDI) mass spectrometry to identify differences between ovarian clear cell carcinoma and endometrial clear cell carcinoma [41]. They identified around 53 candidate markers, including vimentin, Annexin A4, and 14-3-3 beta/alpha, that were differentially expressed between these cancer types.

Besides identifying changes in protein expression, proteomics approaches can also be utilized to study post-translational modifications mediating signaling pathways such as phosphorylation, glycosylation, acetylation, ubiquitination, and SUMOylation [71]. Several groups have studied post-translational modification landscapes in rare ovarian cancers. Faratian et al. used a reverse-phase protein array (RPPA)-based phosphoprotein profiling of histological subtypes of ovarian cancer, including serous, endometrioid, clear cell, and mucinous carcinomas, to identify subtype-specific activation states of druggable oncogenic pathways [42]. The profiles indicated novel therapeutic regimens such as MAPK-inhibition in serous and clear cell carcinomas. Another study aiming to identify characteristic phosphoprotein markers for ovarian clear cell adenocarcinoma (CCA) carried out comparative phosphoproteome analysis using CCA and non-CCA cell lines [72]. The study found that phosphopeptides of SWI/SNF chromatin remodeling/tumor suppressor proteins, including ARID1A and BRG1A, were hypophosphorylated in CCA cells. Targeted mass spectrometry of phosphopeptides validated Ser1452 phosphorylation of BRG1A as a potential CCA marker. Suh and colleagues characterized phosphorylation sites of nuclear FOXL2, which frequently undergoes mutation at c.402C → G (C134W) in granulosa cell tumors, a form of rare ovarian cancer [73]. Using a proteomics approach, the authors identified differential phosphorylation sites at Ser33, Tyr186, and Ser238 that could serve as biomarkers for granulosa tumors. Glycosylation is an important post-translational modification. Secreted and cell surface proteins are often glycosylated, and therefore, glycosylated proteins could serve as potential biomarkers for cancers. Sogabe and colleagues used a dual approach of lectin microarray and isotope-coded glycosylation site-specific tagging (IGOT) LC/MS analysis to identify differential biomarkers for EOCs using cell lines and ascites fluids [74]. A total of 144 glycoproteins were identified for EOC, of which WFA-bound (Wisteria floribunda lectin) ceruloplasmin was found to be a novel biomarker for EOC.

### 3.4. Exploration of the Rare Ovarian Cancer Metabolome

Metabolomics refers to the study of the metabolites/small molecule complement in a biological system [75]. Owing to the development of high-throughput technologies for identifying and quantifying metabolites and their activities, the metabolome is now more accessible to the researcher than ever before. Since metabolites, like proteins, are drivers of cellular processes, the metabolome is gradually being accepted as the ultimate destiny for the flow of biological information. Metabolomics experiments are primarily carried out using nuclear magnetic resonance (NMR) spectroscopy [76] and mass spectrometry (MS) [77]. The most commonly used mass spectrometry-based approaches include Gas chromatography coupled with mass spectrometry (GC-MS) and liquid chromatography coupled to mass spectrometry (LC-MS). Tandem mass spectrometry (LC-MS/MS) is now being increasingly used to carry out metabolomics experiments. Metabolomics can either be carried out in a targeted or untargeted manner [77]. Metabolomics is being increasingly used to delineate molecular mechanisms of various diseases, including cancers. In the context of rare ovarian cancers, there are currently a few studies published.

A mass spectrometry-based metabolomics approach was used to identify potential biomarkers specific to ovarian EOCs [78]. Metabolite markers including N′-Formylkynurenine, Phytosphingosine, and markers belonging to Ganglioside, Lysophospholipids, and Ceramides classes were found to be differentials for EOCs and suggested to be potential biomarkers. Garg and colleagues used a combination of NMR and MS approaches to profile the tissue metabolome of high-grade and low-grade ovarian EOCs [43]. Metabolites belonging to the ascorbate and aldarate metabolic pathways were significantly altered between these EOCs and could serve as potential biomarkers for differential diagnosis. Another study employed an NMR-based metabolomics approach to identify metabolic signatures that could differentiate between controls, platinum-sensitive, and platinum-resistant ovarian EOCs [79]. These signatures serve as potential markers for the prediction of chemotherapeutic response. Global and targeted metabolite profiling of plasma samples from serous ovarian cancers and serous benign controls resulted in the identification of 34 differential metabolites [44]. Evaluation of identified lipid metabolites in combination with CA125 using a model was able to differentiate between serous ovarian cancers and benign controls with high accuracy. Another study sought to delineate the role of HNF1β (hepatocyte nuclear factor 1 homeobox B), a protein exclusively found to be expressed in OCCC and not in other ovarian cancers [78]. Metabolomics of HNF1β_shRNA-stable cell lines found that HNF1β altered cellular metabolism to enhance aerobic glycolysis, causing the “Warburg effect”, which in turn, allowed OCCC cells to survive under stress such as hypoxia.

## 4. Multi-Omics Dataset Integration towards a Systems Biology View of Rare Ovarian Cancers

**Figure 1:**
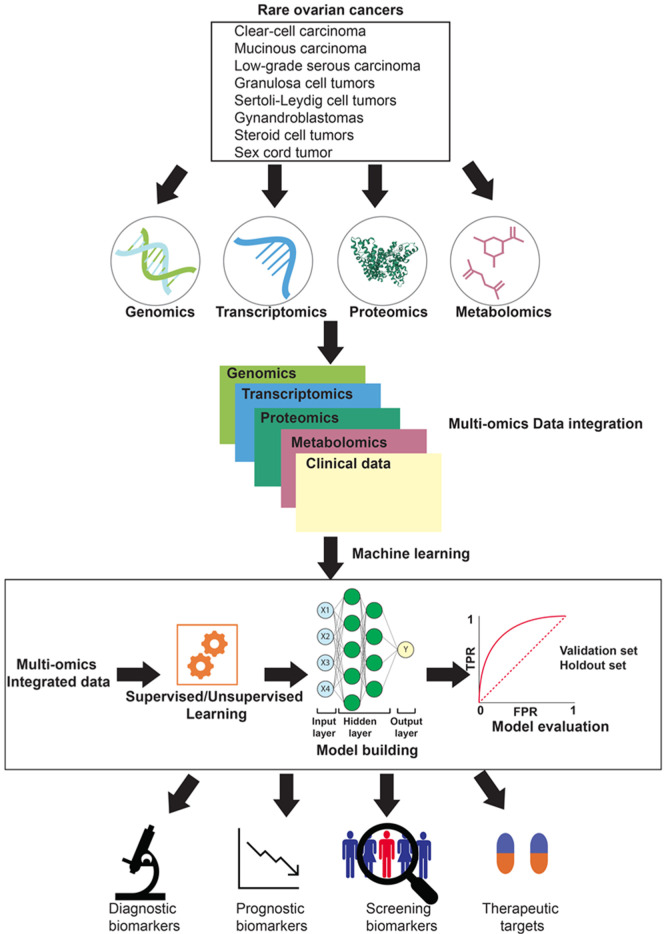
An overview of the various omics approaches for the characterization of rare ovarian cancers and their potential applications. Multiple omics approaches, including genomics, transcriptomics, proteomics, and metabolomics, can be employed to study rare ovarian cancers. Omics datasets from these approaches could be then integrated. The integrated data could then be analyzed using machine learning algorithms to identify diagnostic biomarkers, prognostic biomarkers, screening biomarkers, and therapeutic targets.

Cancer is a complex disease involving alterations in DNA, RNA, protein, and metabolite levels. Genomics, transcriptomics, proteomics, and metabolomics are complementary approaches that enable understanding molecular mechanisms of cancer signaling in considerable detail. The levels of DNA, RNA, protein, and metabolites are largely associated [7], and therefore studying the relationships between the various omics datasets is required. Owing to these reasons, the integration of multi-omics datasets is a significant step towards obtaining a complete view of the molecular mechanisms operating within a cell. However, these diverse omics datasets may not always have linear relationships. Examples of non-linear signaling processes include pathway cross-talk, transcriptional regulatory networks, miRNA-mediated network interferences, feedback loops, spatial regulation of signaling intermediates, the impact of components with varying concentrations, and chromatin-based global expression control [80]. Multi-omics data integration can also help in deciphering non-linear processes in addition to linear processes. In addition, multi-omics data integration is a prerequisite step for analyzing omics datasets using machine learning algorithms [81]. Several computational approaches exist that can help integrate multiple omics datasets. However, the detailed discussion of these approaches and their pros and cons are beyond the scope of this review and have already been review by Duan et al. and Subramanian et al. [82,83]. Multi-omics data integration can lead towards the identification of diagnostic biomarkers, prognostic biomarkers, screening biomarkers, and potential therapeutic targets for rare ovarian cancers (Figure 1).

Proteogenomics is another approach that integrates proteomics data with genomics and transcriptomics data and has been extensively used in cancer biology [84]. Proteogenomics can potentially identify mutant proteins, alternative splicing sites, and fusion proteins or provide evidence of translation for ncRNAs/pseudogenes. These could then be used as potential biomarkers or therapeutic targets for various diseases, including cancers. Proteogenomics has been widely used by various groups around the world to delineate the molecular mechanisms of multiple cancers, including breast [85,86], glioblastoma [87], high-grade serous ovarian cancer [88], colon, and rectal cancers [89], among others. There are currently a limited number of proteogenomics studies focusing on rare ovarian cancers. A proteogenomics approach using reverse-phase protein arrays and mutation analysis identified significant differences between OCCC and ovarian endometrioid carcinomas [90]. However, there are no studies pertaining to proteogenomics of other rare ovarian cancers, and therefore, this could open up novel avenues for exploring the fundamental mechanisms of these cancers.

Recently, machine learning is being extensively used to model disease mechanisms in cancers to identify potential biomarkers and therapeutic targets. Several machine learning approaches have been used to date, including unsupervised, supervised, network-based, deep learning approaches. There are relatively few studies using machine learning to explore the molecular mechanism of ovarian cancers. Kawakami and colleagues used seven supervised machine learning classifiers to predict multiple clinical parameters pertaining to EOCs and thereby were able to differentiate them from benign ovarian tumors with high accuracy [91]. Another study by Elias and colleagues carried out neural network analysis on ncRNA data from EOCs to produce an algorithm to diagnose EOC [92]. A deep learning-based approach has also been used to analyze multi-omics data from ovarian cancers and identify subtypes [93].

## 5. Potential Applications of Omics Approaches to Study Cancer Recurrence and Drug Resistance

Ovarian cancer recurrence is generally not curable, and thus treatment goals shift to include quality as well as quantity of life [13]. Recurrent ovarian cancer is associated with a high symptom burden, both in the number and severity of symptoms [94]. The usual manifestations of recurrent ovarian cancers are pelvic masses in the surgical bed, peritoneal carcinomatosis, retroperitoneal lymph node metastases, pleuropulmonary metastases, and hepatic metastases, however, extrahepatic recurrence may also occur [95]. The standard approach for treating recurrent ovarian cancer is chemotherapy and surgery remains an option for individual patients who should be carefully selected.

Resistance to anticancer drugs, whether intrinsic or acquired, is the major cause of relapse and mortality [96]. Drug resistance may occur due to various reasons, including genetic mutations and/or epigenetic changes. A better understanding of these underlying mechanisms will facilitate the development of novel therapeutic strategies, possibly leading to better clinical outcomes [97]. In this regard, the use of high-throughput genomic, proteomic and functional analytical approaches has resulted in the identification of novel genes and signaling networks that are involved in determining the tumor-responsiveness to a particular therapy.

Genetic heterogeneity has limited the development of targeted therapies for ovarian cancer, which have otherwise been successful in other cancers such as trastuzumab, Herceptin^®^ (for breast cancers with HER2-amplifications), imatinib mesylate, Gleevec^®^ (for BCR-ABL fusion in chronic myelogenous leukemia (CML) or KIT mutant gastrointestinal stromal tumor), amongst others. However, a detailed understanding of genetic events such as aberrations in homologous DNA repair pathways eventually led to the development of poly (ADP-ribose) polymerase (PARP) inhibitors for treating ovarian cancer [98]. Genomic alterations also contribute to the dynamic cell growth and frequent genomic alterations and gene expression changes that contribute to the adaptation to therapy. High-grade serous ovarian carcinoma (HGSOC), carcinosarcoma, and undifferentiated carcinoma are initially chemosensitive, however, as a result of their highly proliferative activity and defects in DNA repair capacity, chemoresistance often emerges [99].

Recent genome-wide studies have elucidated the mechanisms underlying chemoresistance, and these may potentially lead to new therapeutic strategies that target these pathways [100,101]. However, these new approaches may improve progression-free survival, while they are less likely to affect overall survival, underscoring the importance of early detection. [99]. Existing treatment strategies based on the platinum-free interval in recurrent ovarian cancer have been largely driven by the activity of chemotherapy found in HGSOC and endometrioid (HGSEC) histologies. In rarer subtypes, chemotherapy may be less effective, and clinical trials with targeted therapy may be an appropriate strategy. Several studies have been conducted to test the efficacy of MEK inhibitors in LGSOC. MEK inhibitors show a 15–20% response rate compared with 5% of chemotherapy [102]. Treatment with platinum and taxane-based chemotherapy is generally applied post-operatively in patients with advanced ovarian cancer. One way that platinum resistance can occur is through drug inactivation by metallothionein and thiol glutathione, which activates the detoxification system. Changes in the levels of apoptosis-related proteins could also result in drug resistance [103]. For example, tumor suppressor protein p53 (TP53) promotes apoptosis in response to chemotherapy. TP53 is mutated in many cancers, and when mutation or deletion of this gene renders it non-functional, drug resistance can follow. Alternatively, the inactivation of TP53 regulators—caspase-9 and its cofactor, apoptotic protease-activating factor 1 (Apaf-1)—can also lead to drug resistance. [103]. Omics approaches can help decipher the molecular mechanisms of drug resistance and cancer recurrence.

## 6. Conclusions and Future Perspectives

Omics approaches are now considered the standard approaches for the high-throughput investigations of molecular mechanisms of diseases. Besides their utility to discover lead candidates for diagnosis, prognosis, and therapy, there is immense scope of using multi-omics approaches in the clinic to cater to personalized medicine. In several parts of the world, molecular testing is increasingly used in the clinic to diagnose cancer or determine therapeutic strategies [104]. Omics approaches could help delineate molecular mechanisms for rare ovarian cancers and could have a profound impact on therapy, diagnostics, and prognostics. In the future, there probably could be standardized molecular testing for these neglected cancers. In addition, omics approaches can define molecular classifiers of therapy response and identify specific molecular subtypes and assess the efficacy of therapies for these subtypes.

Multi-omics could provide relevant information from different facets and provide a better understanding of rare ovarian cancers. However, several approaches exist for multi omics data acquisition, analysis, and integration, and standardization of these practices are prerequisites for their potential utility in the clinic. Several of these workflows are tedious, and making them more straightforward for their application in the clinic will take several years before they are considered standard investigations. Future developments in sequencing and mass spectrometry technologies need to focus on delivering results faster and cheaper on a large scale and on the miniaturization of equipment.

## Figures and Tables

**Table 1:** A partial list of published omics studies on rare ovarian cancers.

Omics	Study Type	Ovarian Cancer Subtype	Findings	Ref
Genomics	Whole-genome sequencing	Ovarian clear cell carcinoma	prevalent mutations in *ARID1A* (42%), *PIK3CA* (35%), *HLA-DRB1* (25%), *MUC4* (22%), *ZNF717* (22%), and *ARID1B* (18%)	[34]
	Exome sequencing	Mucinous ovarian cancers	mutations in *KRAS*, *BRAF*, *CDKN2A*, *TP53*, *RNF43*, *ELF3*, *GNAS*, *ERBB3*, and *KLF5* genes	[35]
	Targeted sequencing	Low-grade serous ovarian cancers	prevalent mutations in *KRAS*, *BRAF*, and *NRAS*	[36]
Transcriptomics	RNA-Seq	Ovarian clear cell carcinoma	*CPNE8* is a differential marker to distinguish between OCCC and HGSOC	[37]
	Gene expression	Ovarian granulosa cell tumors	24 genes differentially expressed between early and advanced granulosa cell tumors	[38]
	Single-cell RNA-seq	Serous epithelial ovarian tumors	sixteen distinct cell populations with specific cells correlated to high-grade, low-grade, and benign tumors	[39]
Proteomics	2D-gel electrophoresis and mass spectrometry	Clear cell adenocarcinoma and mucinous adenocarcinoma	distinguishing markers between clear cell adenocarcinoma and mucinous adenocarcinoma	[40]
	matrix-assisted laser desorption/ionization (MALDI) mass spectrometry	Ovarian clear cell carcinoma	Differential proteins between ovarian clear cell carcinoma and endometrial clear cell carcinoma	[41]
	reverse-phase protein array (RPPA)	4 histological subtypes of ovarian cancer	subtype-specific activation states of druggable oncogenic pathways	[42]
Metabolomics	Nuclearmagnetic resonance (NMR) and mass spectrometry	EOCs	Differential markers between high-grade and low-grade ovarian cancers	[43]
	Mass spectrometry	Serous ovarian cancers	Differential markers between serous ovarian cancers and benign tumors	[44]

**Table 2:** A compilation of genomic alterations identified in rare ovarian cancers.

Rare Ovarian Cancer Types	Rare Ovarian Cancer Subtypes	Characteristic Genomic Alterations
Epithelial cell tumors	Low-grade serous carcinoma	K/N-RAS (75%), BRAF (20%)
	Mucinous carcinoma	KRAS (50%), TP53 (26%), MSI (20%), HER2-amp (20%)
	Clear cell carcinoma	ARI1D1A (55%), PI3K pathway (PTEN loss PI3KCAmut AKT2ampl), HER2 amp (14%), MSI (10%)
	Carcinosarcoma	TP53 (80%), PI3K pathway (PTEN loss, PI3K mutation)
Germ-cell tumors	Dysgerminoma	KIT mutation (25%), High CNV 11p gain (60%)
	Yolk sac tumor	DICER 1 (10%), High CNV
	Immature teratoma	Few CNV
	Other: choriocarcinoma, embryonal and mixed carcinoma	Few mutations
Sex cord stromal tumors	Adult granulosa	FOXL-2 (missense mutt 402C ˃ G C, >95%, pathognomonic) ER+, PR+, AR+, AKT1/2, Pi3Kca, TGFb
	Juvenile granulosa	GSP mutation (30%), AKT1
	Sertoli-Leydig	DICER-1 (90%, including germline mutations), FGFR2
	Small cell carcinoma hypercalcemic	SMARCA4 deficiency part of the SWI/SNF complex (90%), PDGFR1, FGFR1
	Other sex cord stroma tumors	Poorly known

## Data Availability

This review paper does not report any new data.

## Abbreviations

EOCs	Epithelial ovarian cancers
GCT	Granulosa cell tumors
GWAS	Genome-wide association study
HGSOC	High-grade serous ovarian carcinoma
LC-MS/MS	Liquid chromatography tandem mass spectrometry
LGSOC	Low-grade serous ovarian carcinoma
LncRNA	Long non-coding RNA
mEOC	Mucinous epithelial ovarian cancer
miRNA/miR	Micro-RNA
ncRNA	Non-coding RNA
NGS	Next-generation sequencing
OC	Ovarian cancer
OCCC	Ovarian clear cell cancer/carcinoma
OS	Overall survival
PCR	Polymerase chain reaction
PFS	Progression-free survival
RGCs	Rare gynecological cancers
RNA	Ribonucleic Acid
RNA-Seq	RNA sequencing
TKIs	Tyrosine kinase inhibitors
